# Succesful oral desensitization in a teenage boy with severe cow’s milk allergy

**DOI:** 10.1186/2045-7022-3-S3-P122

**Published:** 2013-07-25

**Authors:** ZU Tamay, B Aliyev, F Dilek, N Guler

**Affiliations:** 1 Department of Pediatric Allergy and Immunology, Istanbul Medical Faculty, Istanbul, Turkey; 2 Department of Pediatrics, Istanbul Medical Faculty, Istanbul, Turkey; 3 Department of Pediatrics, Ethica Incirli, Istanbul, Turkey

## Background

Severe cow’s milk allergy can cause life-threatening reactions. Specific oral desensitization (SOD) can be effective and improve quality of life of the children and their families.

## Case report

A fourteen-year old boy was referred to our clinic for follow-up with the diagnosis of cow’s milk allergy. At 3 months of age, he had immediate rash around his mouth and vomiting when he was first introduced with infant formula. Since then, he was on a milk restriction diet. He had three anaphylactic reactions needing emergency visit, and epinephrine injections. His last anaphylactic reaction was 2 months before the referral. His serum specific IgE was 49.8 kU/L.

## Methods

Specific oral desensitization (SOD) was performed according to protocol developed by Longo et al. The protocol consisted of 2 phases; the first phase was applied at hospital and the second phase was continued at home. The first phase was completed when 20 ml pure cow’s milk was achieved. The second phase was applied at home by the parents with gradual increase in milk dose.

## Results

During hospital admission he had two mild reactios needing antihistamines and nebulised adrenaline. He is still at the second phase of the protocol now. He can tolerate 200 ml of cow’s milk now; he can eat yoghurt instead of milk (half of daily amount of milk).

## Conclusion

SOD can be effective and life-saving treatment especially for children with severe cow’s milk allergy to overcome life-threatening episodes of accidental ingestions.

## Disclosure of interest

None declared.
